# Analyses of Long-Term Epidemic Trends and Evolution Characteristics of Haplotype Subtypes Reveal the Dynamic Selection on SARS-CoV-2

**DOI:** 10.3390/v14030454

**Published:** 2022-02-23

**Authors:** Binbin Xi, Yuhuan Meng, Dawei Jiang, Yunmeng Bai, Zixi Chen, Yimo Qu, Shuhua Li, Jinfen Wei, Lizhen Huang, Hongli Du

**Affiliations:** 1 School of Biology and Biological Engineering, South China University of Technology, Guangzhou 510006, China; bixibinbin@mail.scut.edu.cn (B.X.); jdw198910@hotmail.com (D.J.); 201820136048@mail.scut.edu.cn (Y.B.); bizic_chen@mail.scut.edu.cn (Z.C.); quym796@163.com (Y.Q.); 201830880484@mail.scut.edu.cn (S.L.); weijinfen@scut.edu.cn (J.W.); huanglzh@scut.edu.cn (L.H.); 2 Guangzhou KingMed Transformative Medicine Institute Co., Ltd., Guangzhou KingMed Center for Clinical Laboratory Co., Ltd. & Guangzhou KingMed Diagnostics Group Co., Ltd., Guangzhou 510220, China; zb-mengyuhuan@kingmed.com.cn

**Keywords:** SARS-CoV-2, haplotype subtypes, evolution, epidemic trends, Ka/Ks, substitution rate

## Abstract

The scale of SARS-CoV-2 infection and death is so enormous that further study of the molecular and evolutionary characteristics of SARS-CoV-2 will help us better understand and respond to SARS-CoV-2 outbreaks. The present study analyzed the epidemic and evolutionary characteristics of haplotype subtypes or regions based on 1.8 million high-quality SARS-CoV-2 genomic data. The estimated ratio of the rates of non-synonymous to synonymous changes (Ka/Ks) in North America and the United States were always more than 1.0, while the Ka/Ks in other continents and countries showed a sharp decline, then a slow increase to 1.0, and a dramatic increase over time. H1 (B.1) with the highest substitution rate has become the most dominant haplotype subtype since March 2020 and has evolved into multiple haplotype subtypes with smaller substitution rates. Many evolutionary characteristics of early SARS-CoV-2, such as H3 being the only early haplotype subtype that existed for the shortest time, the global prevalence of H1 and H1-5 (B.1.1) within a month after being detected, and many high divergent genome sequences early in February 2020, indicate the missing of early SARS-CoV-2 genomic data. SARS-CoV-2 experienced dynamic selection from December 2019 to August 2021 and has been under strong positive selection since May 2021. Its transmissibility and the ability of immune escape may be greatly enhanced over time. This will bring greater challenges to the control of the pandemic.

## 1. Introduction

With the global spread of SARS-CoV-2, by 3 December 2021, 264,231,311 people had been infected and 5.23 million died (https://coronavirus.jhu.edu/ (accessed on 3 December 2021)), which has caused huge losses around the world. However, we still do not fully understand its molecular and evolutionary characteristics, especially its origins. Recently, a letter mentioned that knowing how COVID-19 emerged is critical for information of global strategies to limit the risk of future breakthroughs [1]. We strongly agree that it is of great importance to understand the origins of SARS-CoV-2 for future responses to sudden infectious diseases. Recent reviews have also discussed, from various aspects, that SARS-CoV-2 could not have possibly evolved in an animal market in a big city and even less likely in a laboratory [2,3] and its adaptive shift can only happen before the onset of the current pandemic and with the aid of step-by-step selection [2]. Our multi-data on molecular evolution and epidemic trends of early SARS-CoV-2 genomes also support that the virus should have existed for some time before it was discovered [4]. Through molecular epidemiological analysis of 16,373 SARS-CoV-2 genomes at the early stage (from December 2019 to 10 May 2020), we identified nine specific mutation sites of high linkage and four major haplotype subtypes, including H1 (Pango lineage B.1), H2 (identical to the reference sequence at the 9 sites, Pango lineage B), H3 (Pango lineage A.1) and H4 (Pango lineage A) [4]. According to the phylogenetic networks of the four major haplotype subtypes, the estimated ratio of the rates of non-synonymous to synonymous changes (Ka/Ks), the detected population size, and the development trends in chronological order of each major haplotype subtype, the most likely evolution hypothesis speculated that H3 and H4 were the earliest haplotype subtypes, which have gradually been eliminated with selection, while H2 was the transitional haplotype subtype in the evolution process, and finally evolved into H1 [4]. Among them, only the H2 and H4 haplotype subtypes appeared at an early stage in China, and H1 was mainly endemic in Europe in January 2020. Whereas in mid-February 2020, all of the four major haplotype subtypes with relatively higher frequencies had already been found in the U.S. and Australia [4]. However, due to the short-time scale of data collection in the previous study [4], we do not know the subsequent epidemic trends of these haplotype subtypes exactly. And there may be more early genomic data that were submitted later which could provide us with more information on the early SARS-CoV-2 evolution. Therefore, a longer-term real-time analysis of epidemic trends and evolutionary characteristics of haplotype subtypes will help us better understand the early evolution and possible future development of SARS-CoV-2.

Through the analysis of the molecular evolution and the epidemic trends of haplotype subtypes, we almost first reported the H1 variant and predicted the relationship between A23403G (D614G) mutation in the *S* gene and the SARS-CoV-2 infectivity [4]. In addition, the haplotype subtypes analysis showed that the H1 variant not only contained A23403G mutation but also contained three other mutation sites (C241T, C3037T, and C14408T), which almost completely linked to the A23403G mutation site. Since the epidemic variants, such as H1 and B.1.1.7, often occurred with multiple linked mutation sites, we have proposed an efficient method of analyses of key mutations, linkage, and haplotype subtypes epidemic trend, and developed them into two automated tools, AutoVEM and AutoVEM2 [5,6]. AutoVEM is specialized in the analysis of SARS-CoV-2 genomes, while AutoVEM2 can analyze genome sequences of any known and unknown viruses in the case of providing the corresponding reference sequence. Through these tools, we further found that there were more distinctive and highly linked mutation sites in H1-2 (Pango lineage B.1.177) and H1-4-1 (Pango lineage B.1.1.7, WHO alpha) variants, indicating that our method can comprehensively reveal highly linked mutation sites of variants. Moreover, they can provide real-time epidemic trends for each haplotype subtype over time. If sufficient clinical information is provided, we can quickly and reliably predict which mutation sites may be associated with infectivity or even pathogenicity through the epidemiological trends, evolutionary characteristics, and specific site composition of various haplotype subtypes.

There are many other studies concerned about the evolution of SARS-CoV-2. However, most of them are based on single-point mutation [7,8,9] or some new strains that are discovered in a short time or a certain region and are focused on the mutations in the *S* gene [10,11,12,13]. However, little research has revealed the molecular and evolutionary characteristics of SARS-CoV-2 based on haplotype subtypes or regions across a long time systematically. With the accumulation of new genomic data in the GISAID database, millions of genome sequences provide us with an opportunity to further understand the molecular and evolutionary characteristics of SARS-CoV-2 in its early stages and during the process of transmission. By using 1.8 million high-quality SARS-CoV-2 genomic data and the AutoVEM2 tool [6], we identified haplotype subtypes of the global SARS-CoV-2 genome sequences in different periods or regions and analyzed the haplotype subtypes’ frequency, the Ka/Ks ratio, substitution rate, epidemic trends, and epidemic duration, which would provide a global landscape of SARS-CoV-2 molecular and evolution characteristics and a comprehensive molecular genetic basis for the relationship between SARS-CoV-2 evolution and infectivity. In addition, we deduced the evolutionary relationship of the major haplotype subtypes and estimated the evolutionary divergence among early SARS-CoV-2 genome sequences around the world to provide some new insights into the early evolution of SARS-CoV-2.

## 2. Materials and Methods

### 2.1. Genome Sequences

SARS-CoV-2 genome sequences, collected between December 2019 and 31 August 2021, were downloaded from the GISAID database (https://www.epicov.org (accessed on 30 September 2021)) [14], resulting in 2,532,297 genome sequences. Genome sequences that met the following criteria at the same time were retained: (1) length of sequence ≥29,000; (2) number of degenerate bases ≤50 and number of unknown bases ≤15; (3) definite collection time and country (or region). Genome sequences were filtered and the SNVs (single nucleotide variations) of the remaining genome sequences were called by the Call module of AutoVEM2 [6] according to the above criteria. Eventually, 1,852,600 genome sequences were kept and a file containing all SNVs of the remaining genome sequences was obtained.

### 2.2. Identification of the Specific Mutation Sites in Different Evolution Periods

According to the high-frequency mutation sites at different stages identified in our previous studies [4,5] and this study, four different haplotype classification patterns were adopted in the present study (Table S1). The nine specific mutation sites were identified at the early stage of the pandemic [4]. The 16 specific mutation sites were identified at the stage when the Alpha variants had not begun to spread [5]. In the present study, the same specific mutation sites (the nine sites and the 16 sites) and study periods were used (December 2019–10 May 2020 for the nine sites and December 2019–31 November 2020 for the 16 sites) to obtain the major haplotype subtypes that were popular at different stages of the pandemic. The Alpha variant began to become popular around December 2020 [15,16], while the Delta variant began to become prevalent around May 2021 [16]. Therefore, in the present study, we calculated the mutation frequencies at each position in the genome and identified the 30 and 33 specific mutation sites which have relatively high frequency (bigger than 40%) and high linkage from the genome sequences collected between 01 December 2020–30 April 2021 and between 01 May 2021–31 August 2021, respectively.

### 2.3. Identification of Haplotype Subtypes and Major Haplotype Subtypes

For the nine specific mutation sites (Table S1), bases at the nine positions of a genome were obtained, arranged according to their positions on the genome, and designated as the haplotype subtype of the genome sequence. Eventually, all of the 1,852,600 genome sequences got their haplotype subtypes. Frequencies of haplotype subtypes for genome sequences collected during the study period, when the nine specific mutation sites were identified (December 2019–10 May 2020), were calculated, and haplotype subtypes with a frequency ≥1% were retained. Frequencies of haplotype subtypes for all genome sequences were also calculated and haplotype subtypes with a frequency ≥1% were retained. In order not to lose the early popular haplotype subtypes, we took the two haplotype subtype sets with a frequency ≥1% together to get the major haplotype subtypes defined by the nine specific sites. For the 16, 30, and 33 specific mutation sites, which were identified at different evolution stages, the same process was also carried out. Finally, we got four sets of haplotype subtypes (Table S1).

### 2.4. Frequency Changes of Different Haplotype Subtypes Over Time

The frequency of different haplotype subtypes of the four groups of major haplotype subtypes in different continents and countries were calculated in terms of months.

### 2.5. Ka/Ks Calculation

Reference gene sequences were separated from the reference genome sequence (NC_045512.2) according to the annotation information. The Ka/Ks and the number of substitution mutations of the coding regions of a genome sequence were calculated via the following steps: (a) each reference gene sequence was aligned to the genome sequence using MAFFT v7.475 (mafft--globalpair--maxiterate 16) [17]; (b) the aligned files were converted to AXT format files using a Perl script (https://gitee.com/liaochenlanruo/kaks_pupline/blob/master/parseFastaIntoAXT.pl (accessed on 8 May 2021)); (c) the AXT files of all genes were merged into one AXT format file; and (d) the Ka/Ks and the number of substitution mutations of the genome sequences were calculated using KaKs_Calculator v2.0 (KaKs_Calculator -m LPB) [18]. The Ka/Ks and the number of substitutions of the coding regions of all the 1,852,600 genome sequences were calculated following the same aforementioned steps.

The mean of Ka/Ks in six continents, 22 countries with the number of genome sequences >10,000 (Table S12), and China, were calculated in terms of months. The Ka/Ks between December 2019 and March 2020 were calculated as a whole, as there were few genome sequences in the early stage of the pandemic. And the Ka/Ks of all haplotype subtypes defined by the four groups of specific sites were also calculated.

### 2.6. Estimation of Substitution Rate

The correlation between the number of substitution mutations and the number of days between the sequence collection time and 30 December 2019 when the reference sequence was collected was tested using the Pearson method. And the substitution rate was estimated through a linear regression model by the scipy package of Python:

(1) y=k×x+m

Therein, y represents the number of substitution mutations of the coding regions of a sequence; x represents the number of days between the sequence collection time and the reference genome collection time (30 December 2019); k represents the estimated substitution rate (bases/day); m represents the intercept of the fitted function and has no biological meaning in the present study.

The substitution rate of all genome sequences and the H1 haplotype subtype in different continents, major countries (or regions) with genome sequences >10,000, and China were estimated by the above method. The substitution rates of other haplotype subtypes were also estimated in the same way.

### 2.7. Phylogenetic Tree of Early SARS-CoV-2 Genome Sequences in Washington

The 58 early SARS-CoV-2 genome sequences in Washington, collected between 19 January 2020 and 29 February 2020, were downloaded from GISAID. Eventually, there were 57 genome sequences after filtering out three genome sequences (EPI_ISL_416462, EPI_ISL_417166, EPI_ISL_430295) with low quality as described above, and adding the 2019-nCoV reference sequence (GenBank accession number NC_045512.2) and the genome sequence (GenBank accession number MN985325) of the first reported case in the United States [19]. The phylogenetic tree of the 57 genome sequences was aligned by muscle [20] and constructed using the Neighbor-Joining method in MEGA X [21] with a bootstrap of 1000 replicates with the nucleotide maximum composite likelihood transition/transversion substitutions model [22].

### 2.8. Estimation of Evolutionary Divergence among Early SARS-CoV-2 Genome Sequences around the World

Global early SARS-CoV-2 genome sequences, collected between 1 January 2020 and 10 February 2020, were downloaded from GISAID, resulting in 887 genome sequences. After filtering out genome sequences with low quality as described above, there were 785 genome sequences. Genome sequences in Wuhan between 19 January 2020 and 29 February 2020 were also collected from GISAID, resulting in 100 genome sequences, and all the 100 genome sequences meet the above filtering criteria. The 123 SARS-CoV-2 genome sequences outbreak on 21 May 2021 in Guangzhou [23] were also downloaded from the National Genomes Data Center (NGDC, https://ngdc.cncb.ac.cn/ (accessed on 2 September 2021)) and filtered. With the addition of the 55 genome sequences of Washington between 19 January 2020 and 29 February 2020, there are 2, 181, 52, 38, 13, 28, 425, 74, 55, 100, 78 genome sequences from Africa, Asia (not including Chinese Mainland), Europe, North America, Oceania, the U.S., Chinese Mainland (not including Wuhan), Wuhan (1 January–10 February 2020), Washington, Wuhan (19 January–29 February 2020), and Guangzhou, respectively.

The evolutionary divergence between sequences was estimated by MEGA X [21]. Specifically, the number of base substitutions per site among every two sequences was conducted using the Maximum Composite Likelihood model [22]. All ambiguous positions were removed for each sequence pair (pairwise deletion option). There were a total of 29,903 positions in the final dataset. Evolutionary analyses were conducted in MEGA X [21]. The pairwise distances within every region, and between SARS-CoV-2 reference were extracted for further analysis.

## 3. Results

### 3.1. The Substitution Rate of SARS-CoV-2 in Different Continents and Countries

To explore the substitution rate of SARS-CoV-2 in different continents and countries, we estimated the substitution rate of the coding region of SARS-CoV-2 by the linear regression model. The number of substitutions of a sequence and the days between 30 December 2019, when the reference sequence was collected and the date when the sequence was collected, displayed a significantly positive relationship and the Pearson correlation coefficient of many countries and regions were bigger than 0.8 (Table S2). Compared with other continents, Oceania has the biggest Pearson correlation coefficient (r = 0.97) (Table S2), suggesting that Oceania (Australia) could probably provide SARS-CoV-2 with a more natural evolution environment with less human interference.

**Figure 1:**
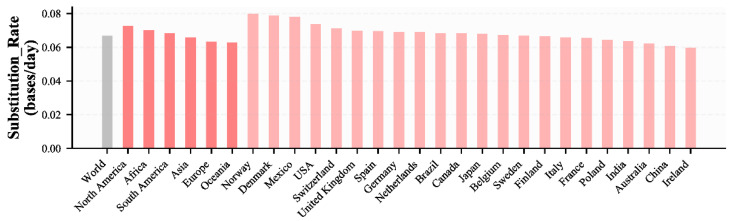
The estimated substitution rate of SARS-CoV-2. The estimated substitution rate of different continents, countries with genome sequences >10,000, and China were plotted, while that of Turkey was not plotted because it had a small Pearson correlation coefficient (r = 0.20).

The estimated substitution rate of SARS-CoV-2 showed that the evolution rate was 0.060–0.080 bases/day in different regions, and the average evolution rate was 0.0688 bases/day all over the world (Figure 1, Table S2). North America has the fastest evolution rate (0.0728 bases/day), while Europe and Oceania have the slowest evolution rate (0.0633 bases/day and 0.0628 bases/day) (Figure 1, Table S2).

### 3.2. Evolution Characteristics of SARS-CoV-2 over Time in Different Continents and Countries

**Figure 2:**
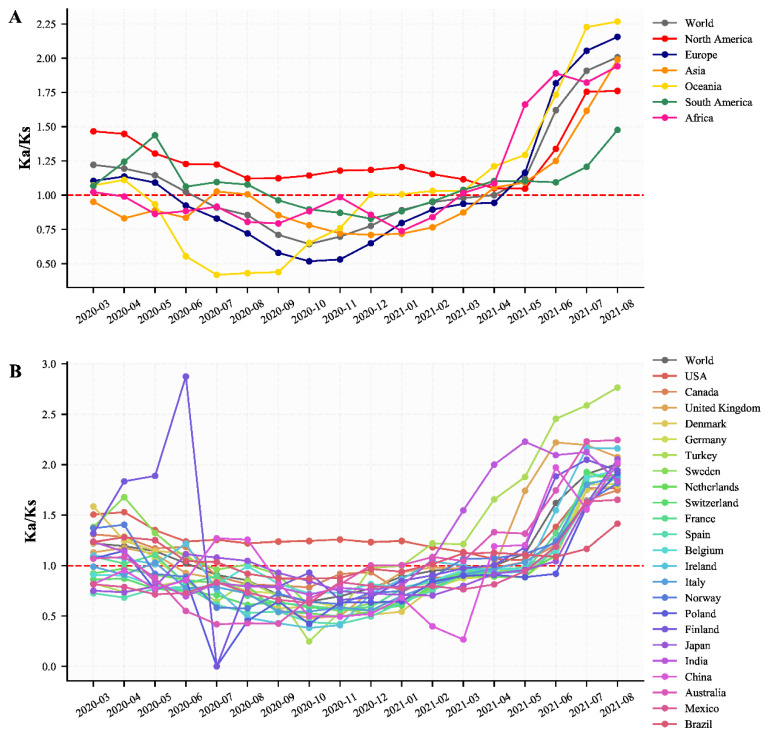
The changes of the estimated ratio of the rates of non-synonymous to synonymous changes (Ka/Ks) of SARS-CoV-2. The Ka/Ks of SARS-CoV-2 between December 2019 and March 2020 was calculated as a whole as there are too few genome sequences in the early stage. (**A**) The changes of Ka/Ks in different continents. (**B**) The changes of Ka/Ks in different countries or regions with more than 10,000 genome sequences and China.

To understand the evolution characteristics of SARS-CoV-2, we analyzed the Ka/Ks of SARS-CoV-2 over time in different continents and countries. The Ka/Ks of SARS-CoV-2 in North America and the U.S. have been always more than 1.0 (1.050–1.761 for North America and 1.054–1.770 for the U.S.) from the early stage to 31 August 2021, indicating SARS-CoV-2 in North America and the U.S. have been under positive selection (Figure 2A,B and Table S3). Those of other continents and countries, except for Poland, Finland, and China, with too few sequences in some months, firstly experienced a large decrease and were smaller than 1.0, especially from July to September in 2020 for Oceania and Australia and from September to December in 2020 for Europe and European countries respectively, indicating that SARS-CoV-2 experienced purifying selection (Figure 2A,B and Table S3). The Ka/Ks then experienced a rapid increase, and by 30 April 2021, it was found that the Ka/Ks of global SARS-CoV-2 were approaching 1.0, indicating that new emerging mutations were neutral mutations and didn’t likely affect the adaptability of the virus. But from May 2021, the Ka/Ks of SARS-CoV-2 of almost all regions experienced a dramatic increase, indicating the SARS-CoV-2 had been under strong positive selection during this period (Figure 2A,B, Table S3). These dramatic changes of Ka/Ks over time indicated the mutations that emerged after May 2021 were beneficial for the virus and likely enhanced the ability of transmission, immune escape, infection, and so on.

### 3.3. Evolution Characteristics and Epidemic Trends of SARS-CoV-2 Haplotype Subtypes

To explore the reason why the Ka/Ks of SARS-CoV-2 displayed such a dramatic change, we analyzed the major prevalence haplotype subtypes of SARS-CoV-2 at different stages of the pandemic. According to the high-frequency mutation sites at different stages identified in our previous studies [4,5,6] and this study, we adopted four different haplotype classification patterns.

**Figure 3:**
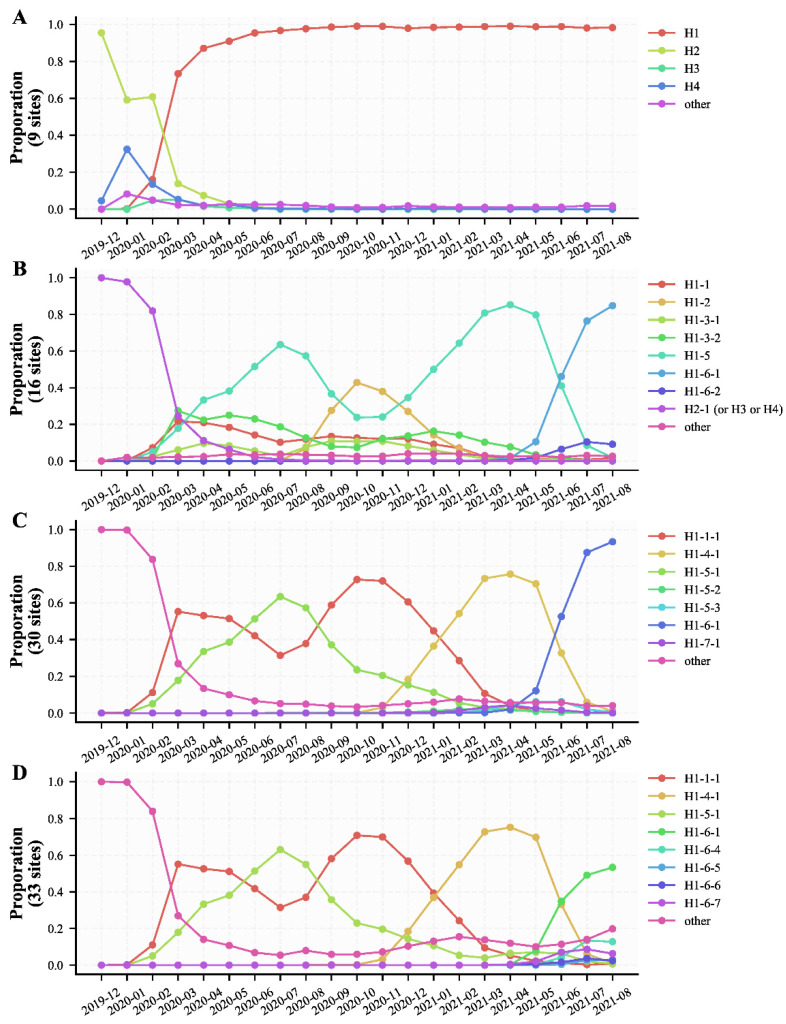
The changes of the proportion of different haplotype subtypes over time. (**A**) Proportion changes of the four haplotype subtypes defined by the nine specific mutation sites. (**B**) Proportion changes of the eight haplotype subtypes defined by the 16 specific mutation sites. (**C**) Proportion changes of the seven haplotype subtypes defined by the 30 specific mutation sites. (**D**) Proportion changes of the eight haplotype subtypes defined by the 33 specific mutation sites.

According to the nine specific sites found in the early stage of the pandemic (December 2019–10 May 2020) [4], a total of four haplotype subtypes with a frequency ≥1% were found, accounting for 98.60% of the total population (Tables S1 and S4). H1 (Pango lineage B.1) had become the most dominant haplotype subtype 2020 around the world by March 2020, while H2, H3, and H4 haplotype subtypes were only popular for a short period at the early stage of the pandemic and gradually disappeared by March 2020 (Figure 3A, Table S5). H3 was detected firstly in the U.S. in a large proportion early in February 2020 (Figure S1). The Ka/Ks of the early haplotype subtypes H3 and H4 were both lower than 1.0, indicating that H3 and H4 haplotype subtypes were under purifying selection (Table S4). Furthermore, their frequencies were both lower than those of the H1 and H2 haplotype subtypes (Table S5), which were consistent with our previous studies [4].

According to the 16 specific sites found between December 2019 and 31 November 2020 [5], a total of eight haplotype subtypes with a frequency ≥1% were found, accounting for 97.09% of the total population (Tables S1 and S4). Except for H2-1 (Pango lineage B) and H1-1 (Pango lineage B.1), all the other six haplotype subtypes (H1-2, H1-3-1, H1-3-2, H1-5, H1-6-1, and H1-6-2) derived from the H1 haplotype subtype (Tables S1 and S4). Among them, H1-3-1 (Pango lineage B.1) and H1-3-2 (Pango lineage B.1) maintained a relatively stable frequency (0.05–0.1 for H1-3-1 and 0.07–0.27 for H1-3-2) worldwide between March 2020 and February 2021 (Figure 3B, Table S5), and they were mainly popular in North America (especially the U.S.) (Figure S2). The Ka/Ks of H1-3-2 haplotype subtype, the dominant prevalence haplotype subtypes in North America (especially the U.S.) between March 2020 and February 2021, was 1.493, suggesting that H1-3-2 has been under position selection and was the reason why the Ka/Ks of SARS-CoV-2 in North America and the U.S. were always more than 1.0 and maintained a relatively stable level between March 2020 and February 2021 (Figure 2 and Figure 3B, Figure S2, Tables S4 and S5). The frequency of H1-5, which derived from H1 and had three more SNPs (G28881A, G28882A, G28883C), exceeded that of H1-1 (H1) from April 2020 (Figure 3B, Table S5). Notably, the frequency of H1-5 experienced a decrease between July 2020 and October 2020, along with an increase in the frequency of H1-2 (Pango lineage B.1.177) (Figure 3B, Table S5). H1-2 was mainly popular locally in Europe and was the dominant prevalence haplotype subtype between October 2020 and November 2020 in Europe (Figure S2). The Ka/Ks of H1-2 haplotype subtypes (0.37 ± 0.097) was the lowest among the eight haplotype subtypes, and the estimated substitution rate was also relatively low (0.0287 bases/day), indicating that H1-2 has been under purifying selection and in a state of slow evolution (Tables S4 and S6). Considering the consistency of H1-2 frequency changes and Ka/Ks changes in Europe, it can be speculated that the decrease of the Ka/Ks in Europe was caused by the popularity of H1-2 (Figure 2A and Figure 3B).

According to the 30 specific sites found between 1 December 2020 and 30 April 2021 in this study, a total of seven haplotype subtypes with a frequency ≥1% were found, accounting for 94.31% of the total population (Tables S1 and S4). There are six haplotype subtypes (H1-4-1, H1-5-1, H1-5-2, H1-5-3, H1-6-1, and H1-7-1) derived from the H1 (H1-1, H1-1-1) haplotype subtype. Among them, the frequency of H1-4-1 (Pango lineage B.1.1.7, WHO alpha) which began to increase from November 2020, exceeded that of H1-1-1 (H1, B.1), and became the dominant prevalent haplotype subtype all over the world from February 2021, and started to decrease from May 2021 (Figure 3C, Table S5). The Ka/Ks of the H1-4-1 haplotype subtype was 0.947 ± 0.180 and the estimated substitution rate was 0.0253 bases/day, indicating H1-4-1 was almost under neutral selection, had a relatively low evolution rate, and was the reason why the Ka/Ks displayed a decrease toward 1.0 in North America and an increase toward 1.0 in other continents between December 2020 and April 2021 (Figure 2A and Figure 3C, Tables S4 and S6).

According to the 33 specific sites found between 1 May 2021 and 31 August 2021 in this study, a total of 8 haplotype subtypes with a frequency ≥1% were found, accounting for 86.41% of the total population (Tables S1 and S4). Among them, the frequency of H1-6-1 (Pango lineage B.1.617.2, WHO VOC Delta), which was first discovered in India, began to increase from May 2021, and gradually replaced the H1-4-1 to become the dominant prevalence haplotype subtype (Figure 3D and Figure S4, Table S5). The Ka/Ks of H1-6-1 (2.095 ± 0.499) was the largest among the 8 haplotype subtypes and far larger than 1.0, indicating the H1-6-1 haplotype subtypes experienced a very strong positive selection (Table S4). And it can be speculated that the dramatic increase of the Ka/Ks of SARS-CoV-2 from May 2021 was the result of the popularity of the H1-6-1 haplotype subtypes throughout the world (Figure 2 and Figure 3D).

The estimated substitution rate of SARS-CoV-2 haplotype subtypes showed H1 haplotype subtype had the highest substitution rate, 0.0671 bases/day, with the r value ≥0.8, suggesting that the H1 haplotype subtype was in a state of rapid evolution (Table S6). And no matter in which haplotype classification pattern, the substitution rate of the subsequent haplotype subtypes derived from the H1 haplotype subtypes is decreasing (Table S6), suggesting that SARS-CoV-2 seems to be more adapted to the host with further evolution, and the evolution rate is gradually decreasing.

The results of haplotype subtype prevalence trends in each country showed that the U.S. had the most complex haplotype subtype populations regardless of the haplotype classification patterns (Figures S1–S4).

### 3.4. Evolution Characteristics of H1 Haplotype Subtype over Time in Different Continents and Countries

**Figure 4:**
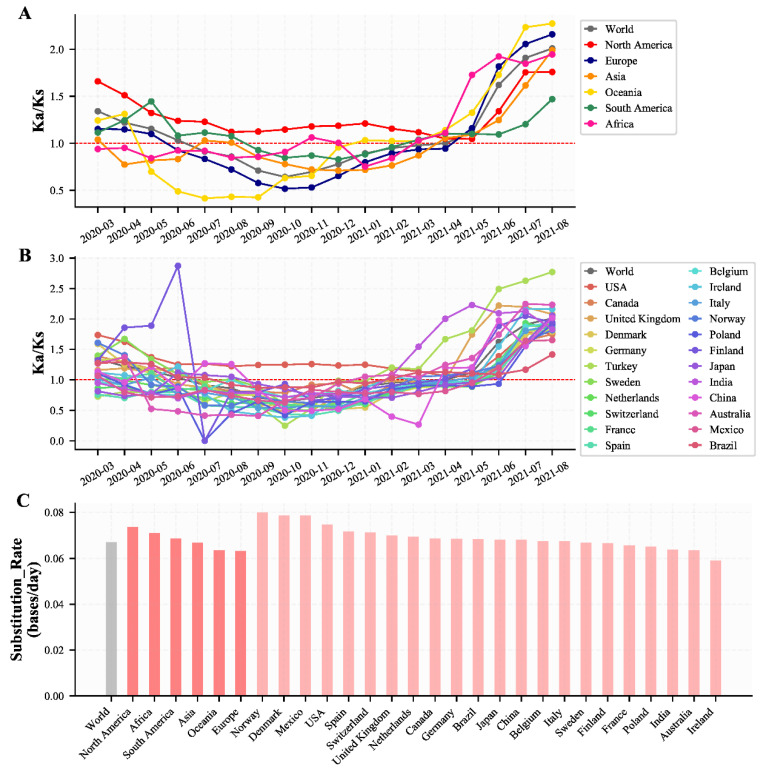
The Ka/Ks and substitution rate of H1 haplotype subtype. (**A**) The changes of the Ka/Ks of H1 haplotype subtype in different continents. (**B**) The changes of the Ka/Ks of H1 haplotype subtype in different countries or regions with more than 10,000 genome sequences and China. (**C**) The estimated substitution rate of the H1 haplotype subtype in different continents and countries or regions. The estimated substitution rate of the H1 haplotype subtype in Turkey was not plotted because it had a small Pearson correlation coefficient (r = 0.17).

According to the haplotype subtypes of the nine specific sites, H1 rapidly became the most dominant haplotype subtype since March 2020 and had maintained its frequency of about 1.0. Therefore, we analyzed the evolution characteristics of the H1 haplotype subtype over time in different continents and countries separately. We found that the Ka/Ks of the H1 haplotype subtype in different continents and countries (Figure 4A,B, Table S7) had similar characteristics to those of all SARS-CoV-2 populations (Figure 2A,B). The estimated substitution rate of the H1 haplotype subtype in different continents and countries (regions) showed that the evolution rate was fastest in North America and slowest in Europe and Oceania (Figure 4C, Table S8), which was also similar to those of all SARS-CoV-2 populations (Figure 1, Table S2). By 30 April 2021, we found that the Ka/Ks of global H1 was also approaching 1.0, and experienced a dramatic increase from May 2021, which was identical to the change of Ka/Ks of global SARS-CoV-2 (Figure 2A,B and Figure 4A,B). Therefore, the H1 haplotype subtype can represent the evolution characteristics of all SARS-CoV-2 populations in different continents or countries since March 2020.

### 3.5. Not All Early Local Cases in Washington State Associated with the Imported Case from Wuhan

**Figure 5:**
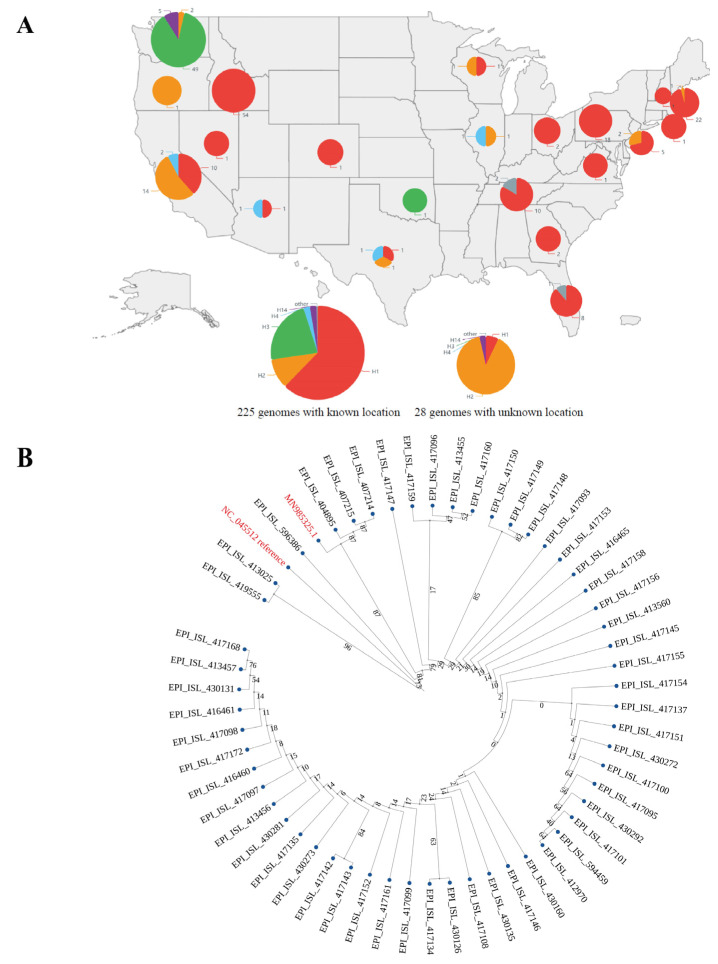
Early haplotype subtype distribution in the U.S. and the phylogenetic tree of early genome sequences in Washington State. (**A**) Genome sequences with the known location were plotted at the corresponding states of the U.S. on the map. (**B**) The reference sequence (NC_045512.2) and the genome sequences (MN985325.1) from the reported first case of the U.S. are marked in red.

Since we found that haplotype subtypes in the United States were very complex from March 2020, H3 appeared firstly in the United States, and H3 was likely the earliest haplotype subtype according to our previous research [4], we downloaded the genome sequences of the United States as of the end of February 2020 (19 January–29 February 2020, 41 days in total) for separate analysis, and found that haplotype subtypes were still very complex and that the state of Washington almost had all H3 haplotype subtype sequences (Figure 5A). Since the first officially reported case in the U.S. occurred in Washington State [19], a rootless evolutionary tree was constructed and evolutionary divergence between genome sequences was estimated by combining SARS-CoV-2 genome sequences of the early cases in Washington state with the reference sequence. Local genomic evolution data in Guangzhou sampled from 21 May 2021 to 18 June 2021 (29 days in total) were used as references to evaluate the evolutionary relationship between the early cases in the State of Washington and the first reported case MN985325.1 [19].

According to the phylogenic tree, at least two genomes (EPI_ISL_413025 and EPI_ISL_419555) in the early cases of Washington state were found to be in completely different branches (Figure 5B). Their evolutionary divergence with the other early case genomes of Washington state ranged from 0.00030 to 0.00070 in 41 days, which were far higher than those between the reference sequence and the other early case genomes in Washington state (0.00010 to 0.00033) in 60 days (Table S9A). This suggested that their evolutionary origin should be different from other early cases, including the first reported case (MN985325.1) in Washington state, because their divergence distances with MN985325.1 were 0.00054 and 0.00030, respectively. The MN985325.1 had four identical genome sequences (EPI_ISL_404895, EPI_ISL_407214, EPI_ISL_407215, and EPI_ISL_596386), and had evolutionary divergences of 0.00007 to 0.00023 with the rest of the genome sequences, which were far higher than those between the first genome GWHBDMN01000001 and the other genomes in May to June 2021 in Guangzhou (0.00003, 0.00007 and 0.00010, of which 66.23% is 0.00003 and 28.57% is 0.00007) (Table S9B). In addition, we found that the evolutionary divergence with the reference sequence increased with time. For example, the average evolutionary divergence between genome sequences in May to June 2021 in Guangzhou and the reference sequence was 0.00122, which was about seven times higher than those of between genome sequences from January to February 2020 in Washington and the reference sequence (Table S9A,B).

### 3.6. Evolutionary Divergence Analysis of Global Early Cases Reveal the Highest Average Evolutionary Divergence in the U.S. and North America and Many High Divergent Genome Sequences

It can be seen from the above data that the evolutionary divergence can represent the evolutionary relationship between the genome sequences of SARS-CoV-2 and the approximate evolutionary time interval. To better understand the evolutionary relationships and evolutionary divergence of early SARS-CoV-2 around the world in the same time interval of 41 days, we analyzed the evolutionary divergence of global SARS-CoV-2 genomic data from 1 January 2020 to 10 February 2020, including Africa, Asia (not including the Chinese Mainland), Europe, North America, Oceania, USA, the Chinese Mainland (not including Wuhan), and Wuhan. The above divergence distances among local genome sequences in Washington State (sampled during 19 January–29 February 2020) and Guangzhou (sampled during 21 May–18 June 2021) were as references (Table S9A–K and S10A–K).

**Figure 6:**
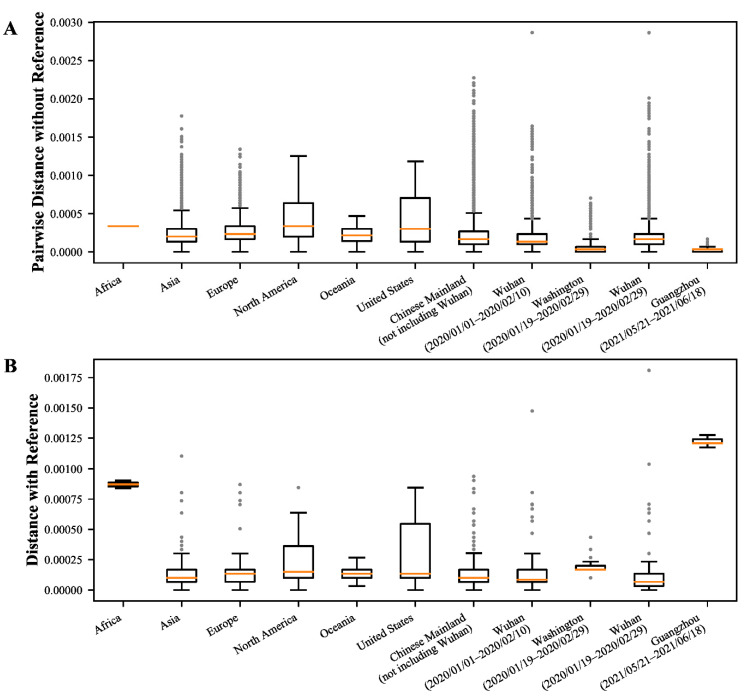
The evolutionary divergence among global early genome sequences. The box extends from the first quartile (Q1) to the third quartile (Q3) of the data, with a line at the median (salmon color). The whiskers extend from the box by 1.5× the interquartile range (IQR). Flier points (silver color) are those past the end of the whiskers. (**A**) The pairwise distance between early genome sequences. (**B**) The pairwise distance between early genome sequences and the reference sequence (NC_045512.2).

As can be seen from the Guangzhou data, the evolutionary divergence of SARS-CoV-2 genome sequences with clear origins transmitted within a short time in a local region is very low (Figure 6A), while in the period from 1 January 2020 to 10 February 2020, we did not observe the phenomenon of the complete low distance between genome sequences in continents or countries or local regions around the world (Figure 6A). The U.S. and North America in particular possess the highest average evolutionary divergence, which is significantly higher than those of Asia (not including the Chinese Mainland), the Chinese Mainland (not including Wuhan), and Wuhan (Figure 6A), indicating that SARS-CoV-2 may have arisen earlier in North America. The obvious evolutionary divergence with reference in Africa may be caused by too few genome sequences (only two) (Figure 6B). Guangzhou’s local genome sequences had the largest average evolutionary divergence of 0.00122 with the reference sequence because their evolutionary divergence time from the reference sequence was nearly one and a half years and far longer than other regions (Figure 6B, Table S9). As of 10 February 2020, there are 40 genome sequences whose evolutionary divergence with the reference sequence is greater than 0.0005 (Table S9A–K). Their high evolutionary divergence was not caused by their genome quality (Table S10A–K), indicating their evolutionary divergence time with the reference sequence might be several months based on the data in Guangzhou, and the genomic data during the early evolution of SARS-CoV-2 was missed.

## 4. Discussion

We performed haplotype analysis using the nine specific sites identified at the early stage [4] and found four haplotypes with a frequency ≥1%, accounting for 98.60% of the total population up to 31 August 2021. The H1 haplotype subtype has become the predominant prevalence haplotype subtype from March 2020. These suggest that the nine specific sites were indeed highly linked and representative at the early stage, and the subsequent variants were mutated from the H1 haplotype subtype. However, the haplotype analysis using the specific sites discovered subsequently showed that there were relatively more haplotype subtypes with a frequency ≥1%, whereas the covered total population is less, indicating that the mutation sites and haplotype subtypes were further complicated in the subsequent stage of SARS-CoV-2 evolution.

According to the four classification patterns, we got some locally or globally dominant prevalence haplotype subtypes and displayed their epidemic trends and possible evolvement relationship. In general, H1 (B.1) appeared at the very early stage of the pandemic and quickly evolved into H1-5 (B.1.1). The population proportion of H1-5 began to increase from February 2020, approached its peak in July 2020, and displayed a decrease between July 2020 and October 2020. When the population proportion of H1-5 decreased, the frequency of H1-2 (B.1.177), which evolved based on H1, experienced an increase and became the dominant prevalence haplotype subtype in Europe between October 2020 and November 2020. The frequency of H1-2 displayed a decrease between November 2020 and April 2021. In contrast to the decreasing trend of the frequency of H1-2, the frequency of H1-4-1 (B.1.1.7, Alpha), which evolved based on H1-5, displayed an increase between December 2020 and April 2021. From May 2021, when the H1-6-1 (B.1.617.2, Delta) began to be popular, the frequency of H1-4-1 began to decrease and almost approached 0.0 in August 2021. The dynamic change of the frequencies of different haplotype subtypes indicates that SARS-CoV-2 is still in a period of rapid evolution, and newly emerging haplotype subtypes likely have competitive advantages over the previous one.

Recently, a new VOC lineage, Omicron, began to become popular around the world [24]. We also downloaded the genome sequences of BA.1 (Omicron) collected between November 2020 and 9 December 2020 from GISAID, and got 737 genome sequences that passed quality control, as described in the methods section. The Ka/Ks of the 737 Omicron genome sequences is 2.206 ± 0.436, which is higher than those of any previous epidemic variants (1.310 ± 0.757 for B.1, 0.370 ± 0.097 for B.1.177, 0.931 ± 0.236 for B.1.1, 0.947 ± 0.180 for Alpha variants, 2.055 ± 0.644 for Delta variants) (Table 1), indicating that the Omicron variant likely has more advantages (such as transmission, infection, or immune escape, and so on) than other variants, especially the Delta variants, and may replace the Delta variant as the next globally dominant variant.

In the present study, we found that the Ka/Ks of the H1-2 haplotype subtype was quite low, 0.370 ± 0.097, and the estimated substitution rate was also relatively low (0.0287 bases/day). These results indicated that the H1-2 haplotype subtype was relatively adaptive to the host, and the specific mutation sites on the H1-2 haplotype subtypes might be related to the adaptation of the virus to the host. The H1-2 haplotype subtype has seven specific mutation sites, T445C in the *ORF1ab* gene, C6286T in the *ORF1ab* gene, C21255G in the *ORF1ab* gene, C22227T in the *S* gene, C26801G in the *M* gene, C28932T in the *N* gene, and G29645T in the *ORF10* gene, that are different from other haplotype subtypes (Table S1). Among them, C22227T, C28932T, and G29645T caused amino acid changes of 222A>V in the S protein, 220A>V in the M protein, and 30V>L in the ORF10 protein, respectively, which may be more closely related to the host adaptability of SARS-CoV-2. The S protein of SARS-CoV-2, as a transmembrane protein, can bind ACE2 with high binding affinity and mediate coronavirus to entry into host cells [25,26]. Recent studies have shown that A23403G in the *S* gene (D614G mutation in the S protein) can promote virus entry into the host cells and enhance the infectivity of host cells [27,28,29,30]. Here we suggest the 222A>V in the S protein may be related to the host adaptability of SARS-CoV-2, and further experimental confirmation is needed. The M protein plays a crucial role in the assembly of SARS-CoV-2, and there is a report that suggests that M protein promotes membrane fusion through binding to the S protein and the host cell surface receptor [31], indicating that the 220A>V mutation in the M protein may be related to promoting viral infection. It is reported that the *ORF10* gene is not essential for SARS-CoV-2 [32]. Therefore, we could not speculate on the association of 30V>L in the ORF10 protein with the host adaptability of SARS-CoV-2. 

Except for North America and the United States, the Ka/Ks of SARS-CoV-2 in other continents and countries showed a significant decrease. This refers particularly to the decrease from September to December 2020 in Europe and the European countries and the decrease from July to September 2020 in Oceania and Australia (Figure 2A,B and Table S3). The Ka/Ks of haplotype subtype H1-2 is 0.370 ± 0.097, which is the lowest one for all haplotype subtypes (Table S4). According to the most prevalent period of the H1-2 haplotype subtype (from September 2020 to December 2020, Figure S2 and Figure 3B), we can infer that the significant decrease of Ka/Ks in Europe and European countries (Figure 2) was caused by the prevalence of the H1-2 haplotype subtype. The large decrease of Ka/Ks from July 2020 to September 2020 in Oceania and Australia could not be explained by the largest proportion of H1 and H1-5 (Figures S1 and S2), as the Ka/Ks of H1 and H1-5 are 1.310 ± 0.757 and 0.931 ± 0.236, respectively (Table S4). Since the SARS-CoV-2 genome sequences in Australia accounted for 91% of the total sequences in Oceania, we analyzed the data in Australia separately. In total, we identified 13 specific sites with a frequency ≥50%, including the four specific sites C241T, C3037T, C14408T, and A23403G of the H1 haplotype subtype and the additional three specific sites G28881A, G28882A, and G28883C of H1-5 (Table S11). Although the frequencies of the other six specific sites are high in Australia (>60%), those frequencies around the world are close to 1% (Table S11), indicating that the prevalent mutation sites in Australia are relatively unique. Therefore, we only analyzed haplotype subtypes and their corresponding Ka/Ks in Australia, and found nine haplotype subtypes with a frequency ≥1%, accounting for 97.91% of the total population in Australia (Table S11). Among them, the proportion of the H1-5-2 (Pango lineage D.2) haplotype subtype was the highest and accounted for 60.32% of the total population in Australia. The H1-5-2 haplotype subtype accounted for almost all proportions in Australia (Figure S5), particularly from July to September 2020. And the Ka/Ks of H1-5-2 is 0.414 ± 0.076 (Table S11), which could explain why the Ka/Ks in Oceania and Australia decreased significantly from July to September 2020.

In the U.S., SARS-CoV-2 maintained a higher Ka/Ks before January 2021, which is completely different from other countries and regions around the world, suggesting that the U.S. may have accumulated its SARS-CoV-2 population, such as the H1-3-1 and H1-3-2 haplotype subtypes. Regardless of the haplotype classification patterns, SARS-CoV-2 haplotype subtypes were far more complex in the U.S. than in any other country since March 2020 (Figures S1–S4), which further verified the previous findings [5], and indicated that SARS-CoV-2 likely evolved longer in the U.S. and that its early evolution data was missed. In addition, among 16 haplotype subtypes detected by February 2020 (Tables S4 and S5), only H3 existed for the shortest time, which further verified our previous observation [4] and indicated that the H3 haplotype subtype, which presented in the U.S., Canada, and Australia at the early stage, was unique and might have existed for a long time when it was detected. If the H3 haplotype subtype disappeared quickly because of its low infectivity, it is difficult to explain why it was detected in the U.S., Canada, and Australia with a relatively higher proportion at the same time at the early stage (Figure S1), while other haplotype subtypes such as H2 and H4 with total frequency <1%, existed for a longer time (Table S4).

There were many evolutionary characteristics of early SARS-CoV-2 haplotype subtypes that did not fit the characteristics of SARS-CoV-2 transmissions, such as that H3 existed for a short time (Table S4), H1, H1-3-1, and H1-3-2 were increased significantly within one month after being detected (Figure 3A, Figures S1 and S2), and that many high divergent genome sequences were found around the world from 1 January 2020 to 10 February 2020 (Table S9C–J). The haplotype complexity of the U.S. was much higher than that of any other countries from early March 2020 to August 2021 (Figures S1–S4), and H3, H1-3-1, and H1-3-2 had a higher proportion of SARS-CoV-2 subtypes from early February 2020 in the U.S. (Figures S1 and S2, Tables S4 and S5). In addition, the U.S. and North America displayed the highest average evolutionary divergence between 1 January 2020 and 10 February 2020 (Figure 6A, Table S9F,H). The proportions of most haplotype subtypes were gradually increased or decreased in the subsequent stage, while such phenomenon was not seen in the early stage. These phenomena all indicated that the genomic data were missed at the early stage of SARS-CoV-2 discovery. In addition, from the perspective of natural evolutionary adaptation and infected population size, SARS-CoV-2 appears well adapted to humans at the very beginning of the pandemic [33,34], which also indicates that the early genomic data during SARS-CoV-2 evolution was missing. Through antibody testing, some recent studies reported positive cases before the first recognized cases in the U.S. or even earlier than the first cases identified in Wuhan [34,35,36]. In addition, SARS-CoV-2 nucleic acid was detected in wastewater samples from Italy in December 2019 [37]. These studies further confirmed the missing of genomic data during the early evolution of SARS-CoV-2 from other perspectives. In this case, it seems difficult to reconstruct the earlier evolution of SARS-CoV-2 based on the current existing genomic data. We don’t even know whether other SARS-CoV-2 subtypes haven’t been discovered at an earlier evolution stage, which is the central issue of the origin of SARS-CoV-2 [2], much less being able to prove that SARS-CoV-2 originated from artificial modifications based on the current SARS-CoV-2 genome sequences and animal coronavirus genomes [38,39]. However, according to the existing genomic data and various analysis results, the U.S. seems to be the place where the virus appeared earlier. As for when and where SARS-CoV-2 specifically originated, we need more coronavirus genomic data of clinical or animal sources from earlier periods before we can make more accurate speculation.

Our research not only focuses on the molecular evolution and epidemic characteristics of SARS-CoV-2 but also provides an idea and method of virus evolution analysis based on linkage and real-time epidemic trends of the haplotype. Compared with focusing on some important genes such as the *S* gene [13], specific site composition and the epidemic trends of haplotype subtypes can provide genome-wide linked mutation information and allow for the evaluation of the relationship between mutation sites and infectivity or pathogenicity or host adaptability more comprehensively. We observed a global decline in the substitution rate of SARS-CoV-2 and that Ka/Ks was close to 1.0 by the end of April 2021, while a dramatic increase of Ka/Ks from April 2021 was observed, which indicated the great dynamic of SARS-CoV-2 evolution. Therefore, all countries and regions around the world should adopt high-throughput sequencing to monitor the evolutionary trend of viruses in real-time, and share the latest viral genomic data as soon as possible, so that global scholars can jointly assess the evolution trend of SARS-CoV-2. In addition, to track the pathogenicity of the mutant strains, genomic data should be integrated with clinical information as much as possible, so that the evolutionary characteristics and infectivity or pathogenicity, or host adaptability of SARS-CoV-2 can be evaluated more comprehensively.

## 5. Limitations

As of mid-February 2022, there were 406 million confirmed cases. However, there were only 8.14 million genomes in the GISAID database, accounting for about 2% of the confirmed cases. Additionally, sequencing data are very limited in low-income countries or regions. Therefore, the non-random sampling of the SARS-CoV-2 population likely caused biases of the result in the present study. In addition, the evolution of SARS-CoV-2 is affected by a multitude of factors including health policies, environmental factors, the viral replication rate, and so on, which were not considered in the present study.

## 6. Conclusions

Through the evolution and epidemic characteristics analysis of 1.8 million SARS-CoV-2 genome sequences spanning 21 months, it has been found that the nine highly linked specific sites identified at the early stage were representative and the subsequent variants were mutated from the H1 haplotype subtypes. The specific site composition of various haplotype subtypes obtained by linkage analysis will provide more valuable mutation sites besides the *S* gene. SARS-CoV-2 experienced dynamic selection from December 2019 to August 2021 and has been under strong positive selection since May 2021. Its transmissibility and the ability of immune escape may be greatly enhanced over time. This will bring greater challenges to the control of the epidemic. We also found some inconsistencies at the early stage, such as the shortest existence of the H3 haplotype, the global prevalence of H1 and H1-5 within a month after being detected, and many highly divergent genome sequences, which indicated that the genomic data during the early evolution of SARS-CoV-2 was missed. 

Although we found the above phenomena and the mutations of epidemic mutants, we could not determine where the mutations of these epidemic mutants came from based on the current methods, and whether they evolved gradually or rapidly under the positive selection. We need supplementary methods, such as monitoring mixed mutations in the intra-host, in order to find the evolutionary traces of epidemic mutants earlier, which might also play an important role in virus traceability research.

## Figures and Tables

**Table 1:** The Ka/Ks of some major haplotype subtypes.

Haplotype Subtype	Pango Lineage	WHO	Ka/Ks *
H1	B.1	NA	1.310 ± 0.757
H1-5	B.1.1	NA	0.931 ± 0.236
H1-2	B.1.177	NA	0.370 ± 0.097
H1-4	B.1.1.7	Alpha	0.947 ± 0.180
H1-6-1	B.1.617.2	Delta	2.055 ± 0.644
Omicron	BA.1	Omicron	2.206 ± 0.436

* The estimated ratio of the rates of non-synonymous to synonymous changes.

## Data Availability

All data relevant to the study are included in the article or uploaded as Supplementary Information.

## Supplementary Materials

The following supporting information can be downloaded at: https://www.mdpi.com/article/10.3390/v14030454/s1, Figure S1: The epidemic trends of the 4 haplotype subtypes defined by the 9 specific mutation sites in different continents and countries (regions) with more than 10,000 genome sequences and China in different months; Figure S2. The epidemic trends of the 8 haplotype subtypes defined by the 16 specific mutation sites in different continents and countries (regions) with more than 10,000 genome sequences and China in different months; Figure S3. The epidemic trends of the 7 haplotype subtypes defined by the 30 specific mutation sites in different continents and countries (regions) with more than 10,000 genome sequences and China in different months; Figure S4. The epidemic trends of the 8 haplotype subtypes defined by the 33 specific mutation sites in different continents and countries (regions) with more than 10,000 genome sequences and China in different months; Figure S5. The epidemic trends of the 9 haplotype subtypes defined by the 13 specific mutation sites found in Australia in different months; Table S1. The specific sites for the four classification patterns and the detailed specific site composition of different haplotype subtypes; Table S2. The estimated substitution rates of different continents and countries or regions; Table S3. The estimated Ka/Ks of different continents and countries or regions in different months; Table S4. Proportion, first detection date, and last detection date of different haplotype subtypes; Table S5. Proportion changes of different haplotype subtypes defined by the four groups of specific mutation sites in different months; Table S6. The estimated substitution rate of different haplotype subtypes; Table S7. The estimated Ka/Ks of H1 haplotype subtype of different continents and countries or regions in different months; Table S8. The estimated substitution rates of H1 of different continents and countries or regions; Table S9. The evolutionary divergence among global early genome sequences in specific continents, countries, and regions; Table S10. The detailed quality information of global early genome sequences of specific continents, countries, and regions for phylogenetic tree construction or evolutionary divergence calculation; Table S11. The detailed information of the specific site and haplotype subtypes in Australia; Table S12. The number of sequences used by each country with sequences >10,000.
