# Supplementary material for: Analyses of Long-Term Epidemic Trends and Evolution Characteristics of Haplotype Subtypes Reveal the Dynamic Selection on SARS-CoV-2
Source: Viruses. 2022 Feb 23;14(3):454. doi: 10.3390/v14030454 (PMC8954678; doi:10.3390/v14030454)
Supplement: Supplementary file 1 [file viruses-14-00454-s001.zip › Supplemental Material_Figures.pdf]

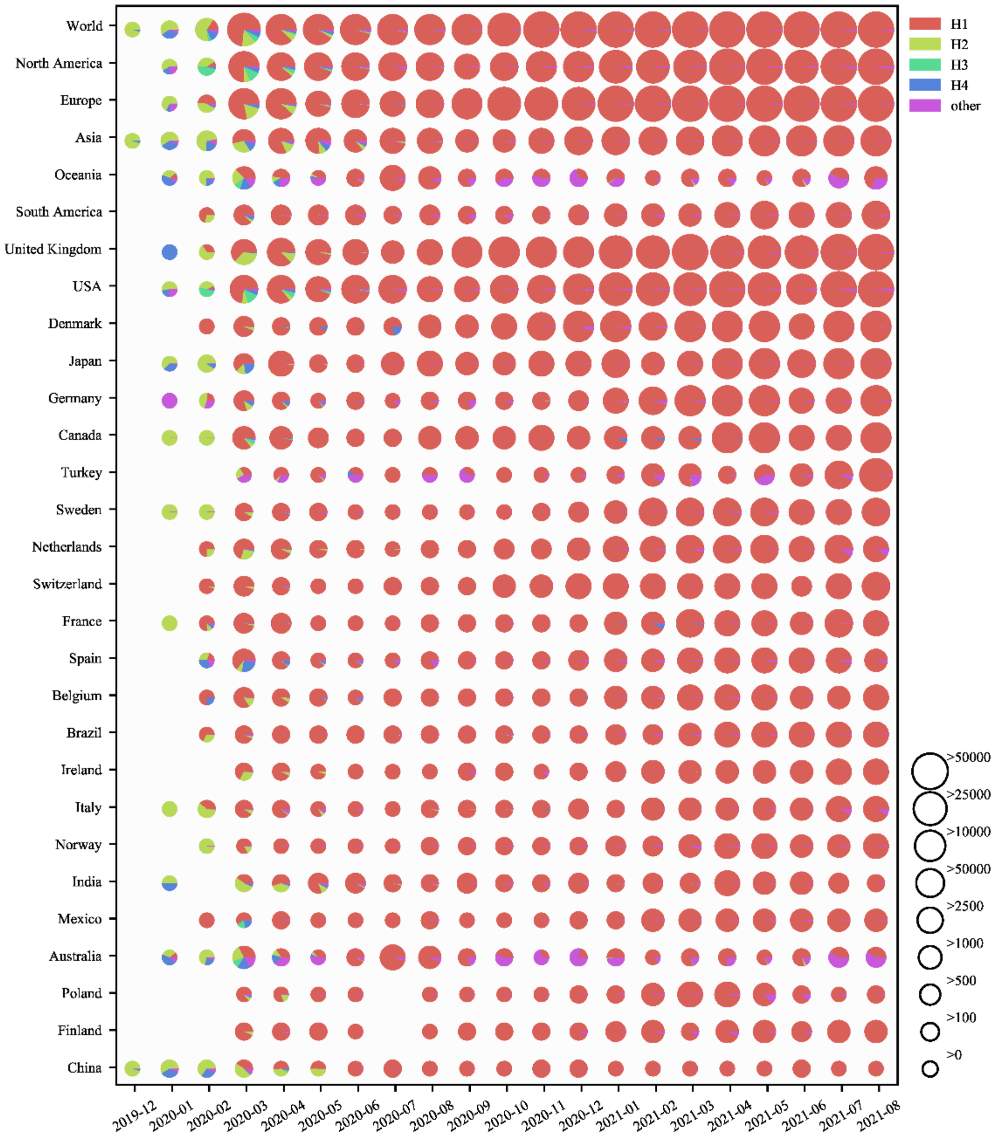

Figure S1. The epidemic trends of the 4 haplotype subtypes defined by the 9 specific mutation sites in different continents and countries (regions) with more than 10,000 genome sequences and China in different months.

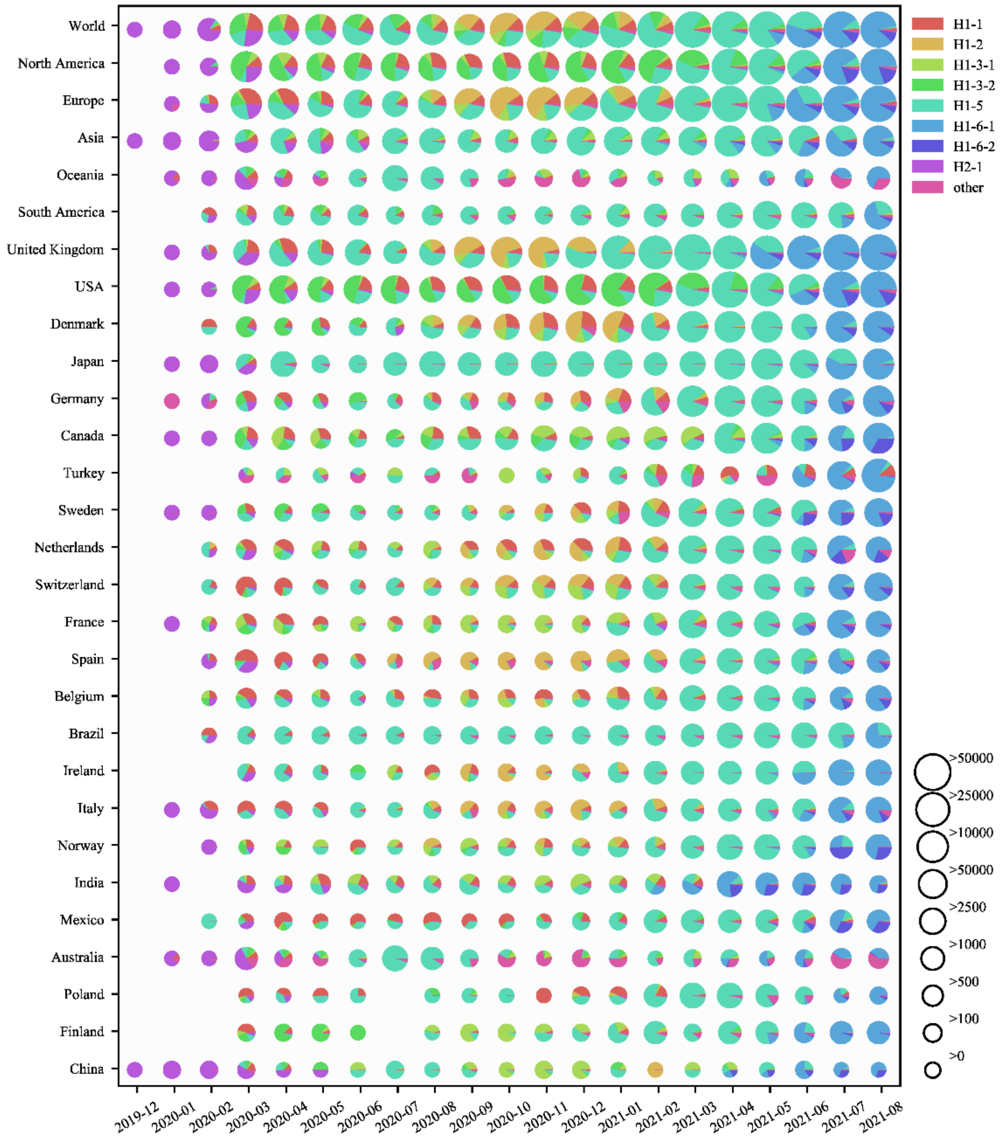

Figure S2. The epidemic trends of the 8 haplotype subtypes defined by the 16 specific mutation sites in different continents and countries (regions) with more than 10,000 genome sequences and China in different months.

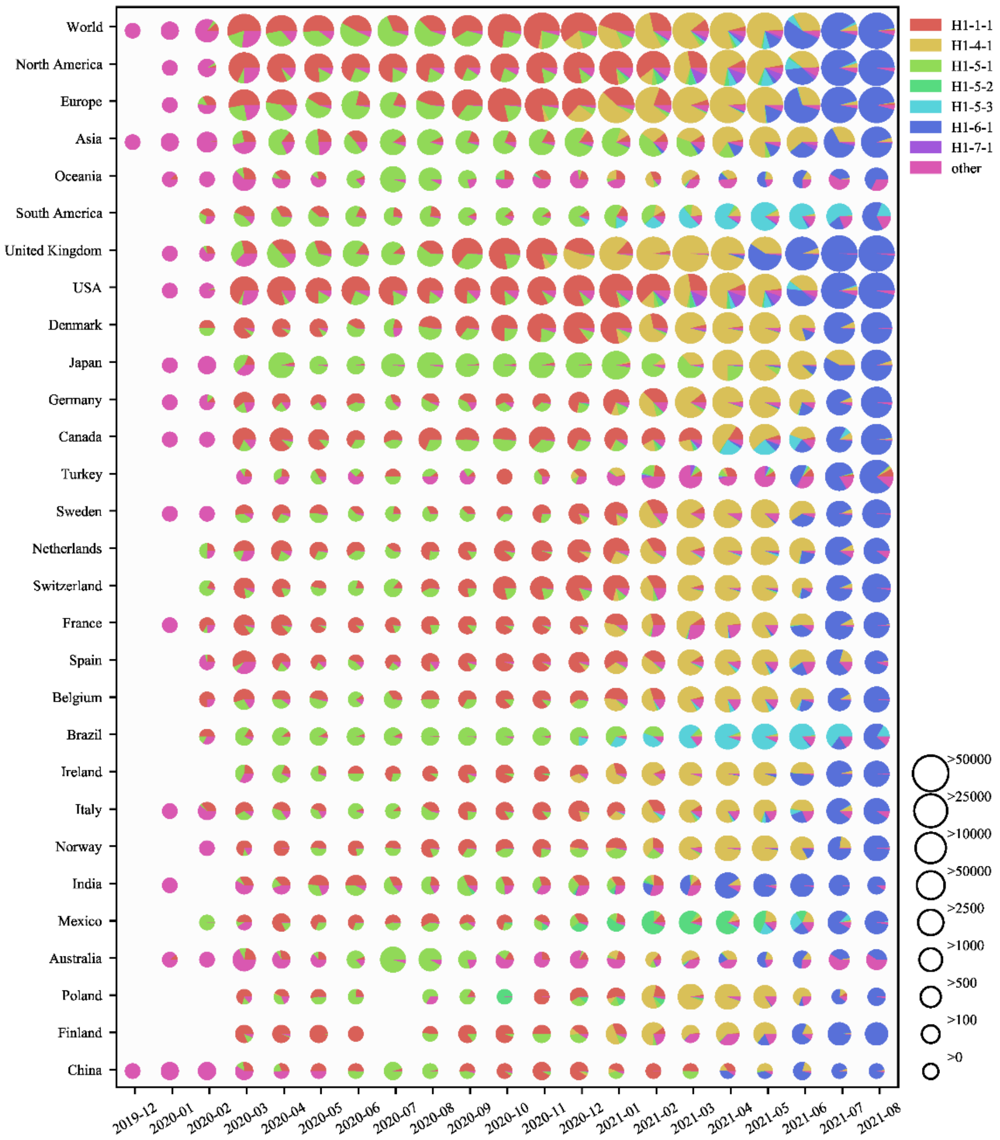

Figure S3. The epidemic trends of the 7 haplotype subtypes defined by the 30 specific mutation sites in different continents and countries (regions) with more than 10,000 genome sequences and China in different months.

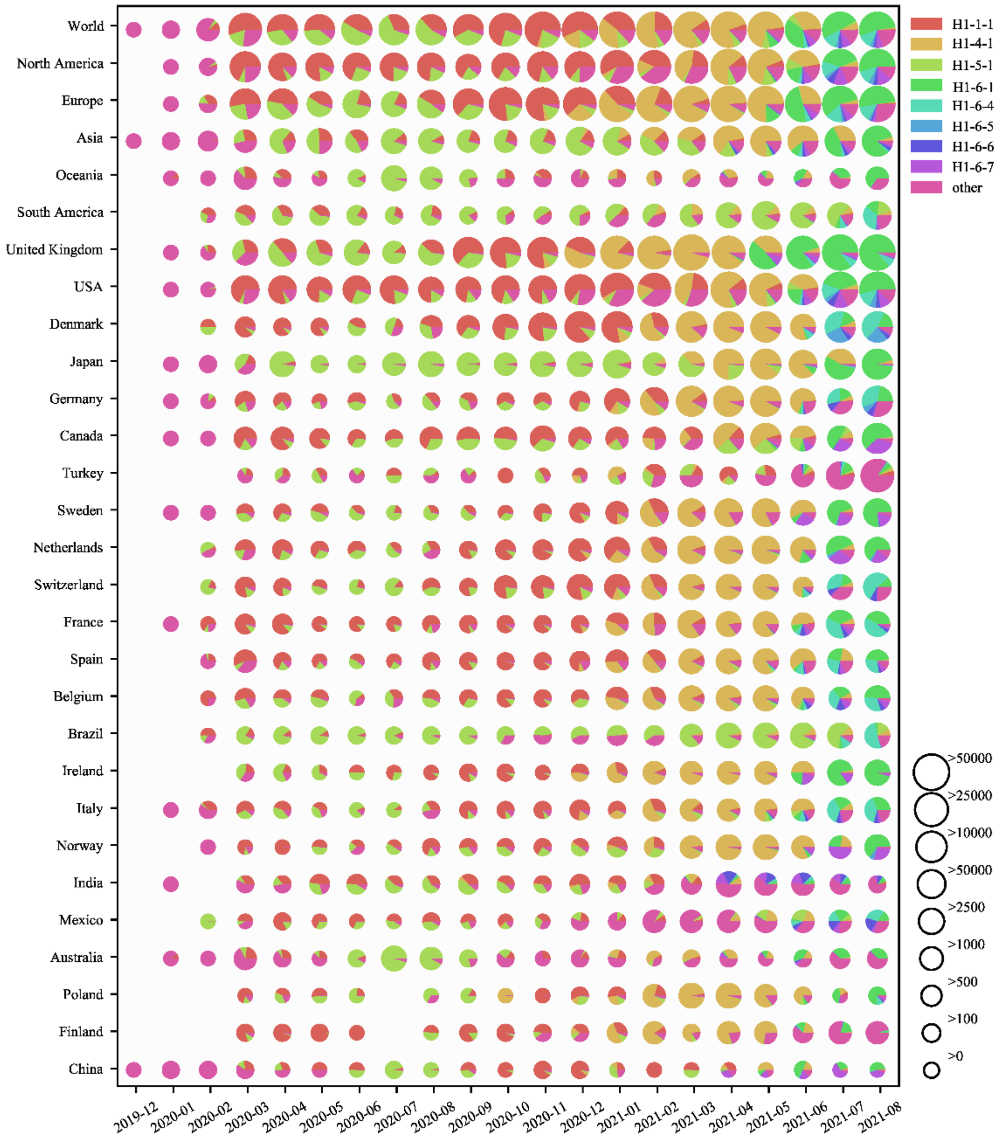

Figure S4. The epidemic trends of the 8 haplotype subtypes defined by the 33 specific mutation sites in different continents and countries (regions) with more than 10,000 genome sequences and China in different months.

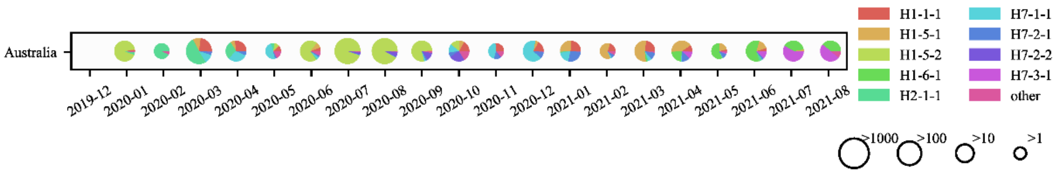

Figure S5. The epidemic trends of the 9 haplotype subtypes defined by the 13 specific mutation sites found in Australia in different months.
